# Brave New Healthcare: A Narrative Review of Digital Healthcare in American Medicine

**DOI:** 10.7759/cureus.46489

**Published:** 2023-10-04

**Authors:** Joseph Pergolizzi Jr., Jo Ann K LeQuang, Ingrid Vasiliu-Feltes, Frank Breve, Giustino Varrassi

**Affiliations:** 1 Operations, NEMA Research, Inc., Naples, USA; 2 Healthcare Policy, NEMA Research, Inc., Naples, USA; 3 Business Administration, University of Miami, Miami, USA; 4 Department of Pharmacy, Temple University, Philadelphia, USA; 5 Pain Medicine, Paolo Procacci Foundation, Rome, ITA

**Keywords:** artificial intelligence, blockchain, cyberchondria, interscatter, telehealth, digital healthcare

## Abstract

The digital revolution has had a profound effect on American and global healthcare, which was accelerated by the pandemic and telehealth applications. Digital health also includes popular and more esoteric forms of wearable monitoring systems and interscatter and other wireless technologies that facilitate their telemetry. The rise in artificial intelligence (AI) and machine learning (ML) may serve to improve interpretation from imaging technologies to electrocardiography or electroencephalographic tracings, and new ML techniques may allow these systems to scan data to discern and contextualize patterns that may have evaded human physicians. The necessity of virtual care during the pandemic has morphed into new treatment paradigms, which have gained patient acceptance but still raise issues with respect to privacy laws and credentialing. Augmented and virtual reality tools can facilitate surgical planning and “hands-on” clinical training activities. Patients are working with new frontiers in digital health in the form of “Dr. Google” and patient support websites to learn or share medical information. Patient-facing digital health information is both a blessing and curse, in that it can be a boon to health-literate patients who seek to be more active in their own care. On the other hand, digital health information can lead to false conclusions, catastrophizing, misunderstandings, and “cyberchondria.” The role of blockchain, familiar from cryptocurrency, may play a role in future healthcare information and would serve as a disruptive, decentralizing, and potentially beneficial change. These important changes are both exciting and perplexing as clinicians and their patients learn to navigate this new system and how we address the questions it raises, such as medical privacy in a digital age. The goal of this review is to explore the vast range of digital health and how it may impact the healthcare system.

## Introduction and background

The ubiquitous and life-changing innovations brought on by the Internet are surprisingly recent. Google was founded in September 1998, Facebook was in 2004, and Twitter was in 2006. Before that, personal computers gained traction in the 1990s and brought with them electronic medical records, which at their inception were hardly more than digital addenda to the old-school paper files [1]. In 2004, a survey from a random sample of healthcare facilities in the United States found that only 13% had a fully functional electronic health records in place and about the same number (10%) did not include any electronic component to their record-keeping system and had no plans to ever implement one [2]. By 2009, US legislation combined with a wealth of new vendors and technological innovations merged to incentivize the transition to all-digital recordkeeping, a move that has or is occurring in other parts of the world as well [1]. In less than a quarter century, the transition to digital healthcare informatics was fully underway led by people, companies, and systems that had no way to envision the goals from the beginning and used by clinicians who do not necessarily see beyond their day-to-day individual interface with these technologies. In some ways, patients may embrace the change more readily than healthcare professionals, as the burgeoning market for smartphone apps and wearable devices indicates.

To some extent, technology has re-invented the patient-provider relationship. The proliferation of health-related information online has engaged and sometimes empowered patients; it has also confounded medical authorities, who have learned that the flow of information across the Internet is challenging even to monitor, much less regulate [3]. As any physician can state from experience, medical information is extraordinarily complex and nuanced, and many factors influence a diagnosis, treatment choices, and prognoses. The lists of symptoms are a mainstay of online health sites, but these can be misleading. Two-thirds of all patients now consult with “Dr. Google” prior to meeting with a clinician, and healthcare professionals have described these patients as everything from “educated” to “misinformed” [4]. Patient support groups proliferate online and serve to bring together people struggling with rare diseases or conditions for which the medical community has few answers. While peer support can be beneficial, the question remains whether patient-to-patient interactions might also have adverse effects. A good example of this are any of several “long COVID” support groups where COVID survivors discussed postviral symptoms and advocated for medical study of these mysterious protracted sequelae [5]. These groups made sure that valuable medical information was available to people seeking it, but did they also encourage self-misdiagnosis and related anxiety [6]?

Twenty-five years ago, no physician would have predicted today’s daily clinical routine of “clicking boxes” or imagined that complex magnetic resonance images or computed tomography scans could be readily digitized and sent easily across the country in moments. The technological revolution is far from over, and the impacts digital healthcare will have on the healthcare system, patients, payers, and clinicians are far from concluded. Indeed, this may only be the beginning. The purpose of this narrative review is to explore many of the novelties and nuances of digital healthcare and to consider how they might affect our healthcare system and what we can do to shape the future rather than be caught off-guard by it. Technology is neither good nor bad; it is rightly seen as a tool that exists for our benefit.

As we navigate these changes, a part of what has changed, along with the technology, is the language we use. Patients no longer get sick; they go on medical journeys. Illnesses no longer run their course; they have a trajectory. Healthcare no longer distinguishes among medical disciplines; it offers a continuum of care. Patients are not just informed; they become empowered. Moreover, in fact, patients are no longer patients at all, but rather healthcare consumers seeking specific services from healthcare providers. The old idea of a doctor meeting a patient in a face-to-face office visit at a clinic is starting to seem quaint, even anachronistic. Regardless of our feelings about renaming or re-imagining the various aspects of healthcare, it is important to understand that these changes describe a different way of looking at healthcare, and it is one that clinicians must proactively manage.

This is a narrative review of various digital technologies of interest to clinicians, patients, and the healthcare industry. The PubMed and Google Scholar databases were searched for various keywords, as were bibliographies of relevant articles. Keywords included "digital health," "cyberchondria," "wearables," "artificial intelligence," "machine learning," "deep learning," "interscatter," "telehealth," "digital twins," "digital trust," and "Dr. Google." This topic is so vast and constantly changing that we made no attempt to systematically review all of the literature but rather provide insights into those topics that seem of most clinical relevance right now.

## Review

The digital revolution has successfully transformed modern-day patients into healthcare consumers with all of the rights and privileges that our society affords to consumers. Consumers not only have specific needs, but they present to the market their list of demands: in general, they want convenience, value, personalized service, and good communication all in a low-cost, easy-to-access system that fits their schedule. Healthcare providers and payers that used to work to improve medical services, outcomes, and patient care are today investing in improving the “consumer experience,” which is really the “patient experience” [7].

A healthcare consumer who wants to take the lead is today able to direct the process of obtaining healthcare rather than relying on physicians to guide them. This, in turn, is leading to what might be called a new “self-management” paradigm, where patients basically make their own care decisions with limited and selective input and involvement of trained healthcare professionals [8]. Pharmaceutical and medical device companies have noticed these shifts and are increasing online services specifically directed to self-managing patients under the rubric of “relational engagement,” where an individual proceeds along a “path” of specific steps for self-care, preventative care, and episodic care [9]. While the shared decision-making model was an important and laudable advancement over the older authoritarian model of a physician as the decision-maker [10], patient self-management is a step further down the road, because the patient has the power to select and limit the involvement of healthcare professionals as treatment choices are made [11]. The original healthcare provider can be shut out of the process if the patient finds those services no longer necessary. With human nature being what it is, this self-management trend is more likely to fragment patient populations than to be widely embraced by all patients. Some patients will whole-heartedly pursue self-management, others will shun it, and many will fall somewhere in the middle. Some have been willing to accept devices and smartphone apps only to find out they have been drawn into a self-managing model whether they wanted that or not [12]. Others become self-managers simply because there are no viable alternatives. Our current healthcare system’s momentum is pushing toward increasing self-management and that means more and more digital healthcare [13]. The following topics are important discussion points in the new digital healthcare system.

Wearables and sharables

Wearable sensors can continuously monitor, record, and track physiologic data, such as heart rate and/or rhythm, blood pressure, oxygen saturation, brain activity, and blood glucose. Modern medicine had years of experience with devices that had integrated sensors, such as pacemakers, diabetes monitors, and implantable loop recorders. Sensors were also used in hospital monitoring systems. Today’s sensors can be detached from devices and worn on the skin in order to wirelessly deliver a steady stream of vital physiologic data. Such wearable sensors can be of value in the hospital setting, but they can also be used by outpatients to monitor their own health. These sensors have been combined with monitors of environmental conditions, such as temperature or humidity, to help people better understand heat indices [14]. While such sensor systems look at physical parameters and vital signs, newer microneedling applications plus sensors are able to view molecular-level changes, such as assessing interstitial fluid for metabolites and electrolytes and tracking their movements [14]. A wealth of sensor-derived data can already be obtained from patients that will continue to expand into the future.

Bidirectional telemetry describes the ability of two or more devices to communicate information back and forth. Bidirectional telemetry may be purely informational (a heart monitor identifies and documents episodes of atrial fibrillation and shares this information with a nurse station) or it may be actionable (an implanted pacemaker adjusts its rate based on perceived levels of patient activity). The next level of this sort of device-to-device communication is interscatter, which forms a part of the so-called “Internet of things” [15]. An example of interscatter is a smart contact lens that is capable of detecting in real time cortisol or cholesterol concentration in tears; these smart contact lenses then send this information to a smartphone and/or to the clinic [16,17]. 

Talking to implanted devices

Beyond smart contact lenses, interscatter, also sometimes called backscatter, is a communications technology that allows medical devices to convert Bluetooth signals from an implanted or wearable in the air to WiFi signals and vice versa. In this way, interscatter creates an opportunity for interactive bidirectional telemetry sharing actionable information among multiple separate systems [18]. In addition, interscatter systems can be deployed to send and receive signals to deeply implanted devices, which previously were difficult for telemetry to access [19]. This overcomes a primary problem with prior-generation radiofrequency telemetry, which required vast amounts of energy to generate low-efficiency signals [20]. Thus, most older-generation devices or sensors with bidirectional telemetry, such as pacemakers, were superficially implanted. Devices or sensors implanted deeply in the body, such as deep within the brain, can now benefit from bidirectional telemetry.

In fact, these new systems need not even know the exact location of the implant. In-Vivo Networking (IVN) is a beamforming technology that uses a multi-antenna system to focus energy toward a deep implant without having its exact location [21]. These devices have been able to identify and communicate with tiny sensors (1 mm) in over 10 cm depths of bodily fluids [22]. The frightening concern or amazing potential of IVN, depending on one’s perspective, is that it can activate passive radiofrequency implantable devices (RFIDs) at distances of up to 38 m [22].

Glucose-monitoring contact lenses have been developed using this technology, which is much less expensive, less cumbersome, and more patient-friendly than conventional glucose monitors. These smart contact lenses monitor tears [23]. Glucose levels in human tears accurately reflect serum glucose, although there may be a 20-minute time lag [23]. A healthcare professional need not monitor data on a daily basis; the contact lenses can communicate the information to a smartphone app, which can then interpret the findings and email remarkable events, as predefined by the physician, to the clinic. When clinicians can predetermine the range of values considered to be of interest, they avoid large streams of essentially unnecessary data.

Artificial intelligence and machine learning

Artificial intelligence (AI) is a broad and complicated field that has powerful reverberations for modern life in general and healthcare in specific. In broad terms, AI is any digital analysis system that is able to perform activities formerly limited to humans, from speech recognition to machine translation all the way to making decisions [24]. Machine learning (ML) is an advanced subset of AI in which the computer “learns” from processing large amounts of data; it does this by recognizing patterns in the information and then building algorithms to better recognize and contextualize those patterns [25]. ML systems do not have to be specifically programmed to learn anything in particular; they gradually over time are able to see and identify trends and events that can be used for predictive analysis. Outside of medicine, ML is possibly best known as the technology behind self-driving cars [26]. The appeal of ML for medicine is that the healthcare system must manage vast quantities of data that are likely to increase exponentially in coming years. Besides clinical data, such as those from electronic medical record data, and research data, the input from patient wearables, guidelines, drug information, diagnostics, and payer data will soon be routinely added. Only ML can collect, consolidate, and organize these numerous inputs to create succinct and coherent reports. ML will be crucial as we advanced toward personalized medicine.

AI has numerous clinical applications, such as gathering information from electrocardiogram (ECG) tracings or analyzing how a specific abnormality may appear on a magnetic resonance image [27,28]. The optimal use of AI in healthcare would be to integrate its powerful data-crunching capabilities under the expertise of human clinicians. An urgent and unmet “medical need” at present is the paucity of big-data scientists dedicated to healthcare applications [29]. For example, drug titration for heart failure patients can be extremely challenging as these patients take multiple drugs and must be cautious about day-to-day changes in their weight, fluid retention, heart rate, heart rhythms, and blood pressure; AI systems may one day be able to track patient wearable monitors and provide daily reports and occasional recommended medication adjustments. It then becomes incumbent on clinicians in terms of how to deploy and optimize such systems without making them a mere observer or irrelevant. AI exists to manage large amounts of data, identify information it is told to look for, and report on data-based patterns. More advanced systems can tease out patterns and report on trends. AI in general should improve the workflow, but it should not function as a substitute for clinical expertise. The clinical team is still the final authority and some AI applications still require considerable human oversight for proper use.

AI is the foundation, but it has been superseded at the next level by machine learning (ML) and eventually by deep learning. The summit of this pyramid of AI at this time is artificial neural networks (ANNs) [30]. ML uses data to find patterns from which it develops algorithms to better identify those patterns and their associations. ML uses subsets of data and various weights and combinations to arrive at highly advanced algorithms, and ML works in four main modes: supervised, unsupervised, semi-supervised, and reinforced learning [30]. ANN is the most advanced form of ML currently available. ANN creates nodes of knowledge that communicate bidirectionally with one another, analogous to neural networks [30]. Deep learning involves complex and multilayered ANNs, which automatically translate data into multiple levels of abstractions. Such deep learning networks of ANNs can be useful in reading medical images or studying genomics [31,32]. Deep learning was recently able to identify two distinct subtypes of glioma from genomic data and offer insights into their individual molecular mechanisms [33]. The system was not commanded to perform this task; it identified the patterns from the data and “taught itself” what to look for and report.

ML algorithms can help harness copious amounts of data by developing systems and algorithms that can interpret the patterns within the data streams, allowing these data to be utilized for clinical or epidemiological applications [14]. This implies that in the not-so-distant future, certain technologies, such as wearables coupled with ML, could be used to diagnose illness and monitor treatment response [14]. This challenges the role of the traditional physician who may be relegated to more of an observer or possibly a referee rather than directing clinical care. Alarming as this sounds, such “automatic diagnoses and treatment” may help the healthcare system of the future provide more care to more people for less money. Another potential benefit is that it could also allow physicians to devote more time to managing complex or highly nuanced cases rather than routine illnesses and conditions.

AI and ML are likewise playing a role in drug development by aiding in drug discovery processes, such as toxicity prediction, peptide synthesis, ligand-based virtual screening, drug monitoring, pharmacophore modeling, and data mining [30]. A more thorough discussion of the role of AI, ML, and deep learning in drug development goes beyond the scope of this review.

A digital twin is a broad term for a digital representation of a physical object, process, or system, designed in such a way that events, tests, and integrations, among others, can be simulated in an effective and meaningful way. The real object or system is connected to its digital twin via data that can then be used to gain information. In this way, digital twins allow for the safe, effective, and cost-effective exploration of real-world systems, although not all of the ethical issues raised by digital twins have been clarified [34]. The design, development, and deployment of digital twins is complex and may involve other complex digital technologies, such as AI, blockchain, and the Internet of things [35]. Digital twins may be of tremendous value in healthcare as an accelerator between research to clinical deployment of medications or procedures. They have the potential to truncate the regulatory pathway of medical devices. They may allow for precision medical solutions, greater product safety, and advanced training experiences for clinicians [36]. Digital twins may be a game-changing albeit disruptive technological innovation for medicine and could drive innovative diagnostic and treatment approaches [37].

Virtual care as a part of the continuum of care

COVID-19 was the great disrupter in healthcare delivery and accelerated the shift away from in-person clinical encounters toward Internet-based care, smartphone applications, and remote care [38]. Of course, the basic foundational infrastructure for virtual care was already largely in place in the United States before the pandemic; it just expanded rapidly during lockdowns [39]. Virtual care solves many old problems: it gives greater access to care for people who live in rural areas, it can maximize limited healthcare resources, and it appears amenable to integration into a variety of other healthcare pathways. Furthermore, virtual care is convenient for patients, reduces travel time and the burden of travel, and imposes less time investment for patients [40]. As the population increases and healthcare resources decrease, telehealth and virtual care may be an important way to leverage benefits to more people.

To be sure, there are numerous issues that revised healthcare delivery via telehealth opens. Training and credentialing healthcare professionals for the unique challenges and limitations of virtual care remains an open issue. For one thing, traditional licensure and credentialing are often location specific (limited to a particular state) while virtual health can be national in scope [41]. There is also the issue of training in that healthcare delivery in the virtual context may require specific training and skills [42]. Furthermore, technological advances mean that virtual care providers need to stay updated on new technologies and systems, which is not always the strong suit or primary interest of clinicians.

Virtual care delivery requires robust platforms that can be integrated into the whole continuum of care, ideally working well for patients, the healthcare system, medical records, and payers, all while preserving confidentiality and protecting patients privacy [43]. In the digital era, medical data storage has already started to migrate away from brick-and-mortar data facilities toward cloud-based solutions. Contemporary cloud systems have more robust data-security systems and cyber controls than earlier systems [44]. However, another important issue emerges: Who owns that data and who controls how data are shared? Are there protections against third-party or fourth-party use of data? Does the patient own his or her own data or does the system control it? Can the system sell or share it? While cloud-based systems seem ephemeral, cloud providers also have a physical presence. At issue is whether they must be in specific locations to service our healthcare systems. For example, can a cloud-based company for medical data be located outside our borders? Are there political risks to locating cloud storage facilities for our medical data in foreign countries?

The rollout of 5G technology ultimately support virtual healthcare because it will create a faster, more reliable Internet [45]. The seemingly continuous technological advances are a fact of life in digital health. Healthcare systems may prefer the familiarity of legacy systems, but eventually these outdated systems cause perilous gaps in healthcare data integrity and privacy protection [46]. One notable challenge in moving to more virtual care is the transitioning of legacy systems to the cloud or other advanced approaches. Cybersecurity is a real consideration for protected health information. In the first half of 2022, there were 337 data breaches involving 19 million health records [47]. Most of these cyberattacks involved third-party vendors, so even if the healthcare records are secure in the cloud, the healthcare data may migrate to less-secure locations [47].

Augmented reality and virtual reality

Augmented reality involves the integration of three-dimensional (3D) virtual objects into a real 3D environment in real time. Thus, augmented reality “augments” or adds virtual element(s) to an otherwise real-world experience. Virtual reality, on the other hand, is an immersive virtual experience usually requiring a headset device. It has been estimated that augmented reality is roughly 25% virtual, while virtual reality is 75% or more virtual [48]. Familiar to many young people in gaming applications, augmented and virtual reality systems are being used for medical education, such as training in dentistry [49], and have also been deployed among surgeons to facilitate planning for complicated surgery [50]. Extended reality devices combined both augmented and virtual reality to offer the full spectrum from absolute reality at one extreme and complete virtuality at the other [51].

New applications for these emerging technologies seem limitless. For instance, they could be used in psychological studies or treatments for patients with phobias. Individuals with cue-based phobias are highly sensitive to visual cues and may be able to unlearn their phobic triggers in a virtual reality setting [52]. Such applications may provide greater insights into the nature and treatment of cue-based phobias. In biomedical engineering, extended reality provides a way to explore 3D models in 3D; this includes models of the human heart to studies of molecular structures [51]. The augmented reality microscope (ARM) from Google combines augmented reality with AI to diagnose cancer, based on real-time microscopic images [53]. The so-called “expansion microscopy” uses virtual reality to enlarge cell structures that are difficult if not impossible to analyze with the use of conventional microscopes [51].

The marriage of telehealth with extended reality has created a new dimension for remote clinical consultations. Using a virtual environment, extended reality telehealth applications might allow clinicians to observe patients as they move, exercise, or play games, so that clinicians can observe and provide diagnostic feedback. This form of remote exercise therapy has been particularly beneficial to disabled individuals who require physical therapy but find in-clinic visits a hardship [51].

While many of these sophisticated technologies require advanced equipment and specialized training, some extended reality tools are becoming more streamlined and integrated into daily life. Google Cardboard, for example, is a virtual reality viewer application that can be used with a smartphone. A Google Cardboard viewer can be purchased at big-box stores or online for a few dollars. This simple cardboard box device allows for stereoscopic viewing with extended reality applications on a smartphone, albeit with certain limitations [51]. It is the low cost and ease of use that makes Google Cardboard so appealing [54]. Higher-end viewers are also available.

The challenges with these systems for healthcare applications are numerous. Some come with a steep learning curve, some are expensive and exorbitant to update, and many present new cybersecurity issues and concerns over patient privacy. In addition, virtual reality has been described as “addicting,” especially when used for entertainment, and tends to favor isolation rather than collaboration [51]. It is not clear if the extensive application of virtual reality systems in healthcare settings would counteract the benefits of collaborative and interdisciplinary approaches to care.

Internet: "Dr. Google will see you … now or whenever"

The Google search engine indexes and serves up millions of websites in response to user queries; it orders these results based on “ranking criteria,” which simplistically means the best matched, most authoritative, and most on-target sites appear first, based on the user’s search terms. Google calls this system PageRank, where Page is the surname of Google cofounder Larry Page [55]. Google ranking algorithms are proprietary, closely guarded, highly secret, and the subject of great interest to hackers and online denizens. Unlocking even part of the algorithm is no easy task, since Google’s algorithms change frequently and without warning.

A study of 21 health-related websites found that since August 2018, Google has been limiting the visibility of these medical sites [56]. Google has the ability to elevate a site’s appearance in search rankings or decrease it, and the company does not disclose why or how this is done. It has been argued that page ranking simply provides the optimal user experience, but some believe that the results may be manipulated for commercial, political, or other purposes [57]. Putting the question of whether that is happening or not to the side, it is possible to use page ranking in this way. Coming up on top at Google is very important; 28.5% of Google searchers click on the very top result of the first page; the 10th result on the first page garners a mere 2.5% clicks, and most searchers do not look beyond the first page of results, although Google routinely serves up hundreds of pages of results [58].

A plausible explanation for the recently observed trend that certain medical sites are being suppressed in Google ranking is the Google methodology that favors sites with “expertise, authority, and trust” (EAT) [59]. It is no secret that the Internet abounds with sites of varying quality. Furthermore, it should be noted that there are no restrictions on website creation: anyone can create and run a medical website. The contention is that following the EAT model, Google is shunting website traffic away from less reputable and toward the more academic medical sites. The problem for site owners is that much of this happens in complete obscurity; medical sites do not know if they are being suppressed or how to do better in search ranking. There is a concomitant Google policy with an acronym name: YMYL. YMYL stands for “your money or your life” (YMYL) and describes sites that offer content and advice related to finance (shopping sites and financial advice sites), medical sites, legal advice sites, and public information about topics, such as social services or disaster preparedness [60]. Google scrutinizes YMYL sites to a much greater extent than other types of sites. Any website that offers medical content and particularly medical advice will fall into the YMYL category. There is very limited guidance for website owners to understand how the Google algorithms evaluate their sites, which can have serious consequences for medical sites.

About one billion health-related questions are posed on Google every day; about 7% of all Google Internet searches have something to do with healthcare, with queries about symptoms, conditions, medications, and insurance the most frequent [61]. More than half of patients seeking medical care for a specific condition have sought online information from “Dr. Google” prior to making the appointment for consultation [61]. In other cases, people use Dr. Google to find information about potentially embarrassing conditions, such as sexual dysfunction [62]. The fact is that Google is used extensively for medical information and appears to be directing people to specific medical sites over others.

Despite the general alarm over online misinformation and “fake news,” there is a great deal of sound, credible, and helpful medical information online that can be used to educate and empower patients. The real key to a beneficial Internet experience requires health literacy on the part of the information seeker, that is, the ability to understand health-related information and act on it [63]. A person with low health literacy will likely not benefit from Dr. Google, even if high-quality sites are consulted. A person with high health literacy can successfully navigate many medical sites of varying quality and discern which information is most reliable. So important is health literacy that it has been recognized as a social determinant of health [64]. The impact that low health literacy can and will continue to exert on the international healthcare system is large and largely under-appreciated [65-69] (see Table 1). Some of the rightful concern about misinformed patients may be more correctly attributed to low health literacy than medical websites of varying quality. 

**Table 1:** Impact of health literacy on healthcare and health education Low health literacy has profound ramifications for all healthcare encounters, but the migration toward more digital healthcare will likely exacerbate these problems caused by an inability to understand and act on health information. All of the ramifications of these issues may result in medications taken improperly and/or lack of adherence [65-69].

Health literacy issue	Ramifications	Possible remediation
Most Americans read at the 8-9th-grade level, but most medical content is written at grade 10 or higher levels.	Adults may be unable to comprehend the material or be unable to properly fill out medical forms. Adults may misunderstand information and think they understand it.	Produce materials written at a 6th-grade level or offer both “advanced” and “simplified” content.
About half of Americans do not understand medical labeling	Adults cannot understand how to take their medications, possible interactions, and risks versus benefits.	Produce simplified labeling or possibly add a prominent “patient-friendly” summary to labeling Infographics or illustrated instructions may be helpful.
About 14% of the US population is foreign-born and 22% of the people in the US do not speak English at home.	Many people will not be able to read or fully comprehend English-language materials.	Translations and/or the use of illustrated instructions.
Older patients may lack technical expertise to go online and/or have visual or auditory limitations.	Older patients often need more and more frequent medical care for comorbid conditions but may be at least able to get information online or process information they are given by healthcare providers.	Large-type printed information, person-to-person consultations. Make allowance for health-related limitations. Make printouts of online information available.
About 25% of parents have low health literacy.	This may affect their ability to make medical decisions for their children or to get proper care for children. It may also lead to poor nutrition.	Make sure parents understand treatment options for their children; use simple language Infographics or illustrated instructions.

Cyberchondria 

Cyberchondria is a condition that can occur when patients use online information to diagnose themselves and then seek medical help. In an online survey of 471 respondents, 40% had symptoms of hypochondria, and those respondents used online resources more and were more likely to deem online medical sources as credible and trustworthy than those without hypochondria [70]. Cyberchondria is being discussed as a psychiatric condition, but it is not clear if it is a form of obsessive-compulsive disorder or whether it relates to anxiety about health [71,72]. From an online survey of 749 respondents, risk factors for the severity of cyberchondria were found to include health-related anxiety, obsessive-compulsive tendencies, and intolerance of uncertainty [73]. Another study found a strong correlation between problematic Internet use in general and cyberchondria in particular [74]. So pervasive is the problem that a cyberchondria severity scale has been proposed to help quantify the phenomenon [75]. A framework for understanding cyberchondria has been proposed, which describes a vicious circle in which compulsive seeking of online health information amplifies health anxiety, which then drives the individual to seek more online information [76].

Blockchain

Blockchain, best known in its application in cryptocurrency, may turn out to be one of the most disruptive and important innovation in healthcare. Blockchain technology is able to take vast amounts of data, keep these data fully anonymous, allow for complete transparency (every user has the potential to see everything), and decentralize these data, which in turn enhances data security. To accomplish these objectives, blockchain serves as a ledger that can never be changed or altered. Every transaction is recorded permanently. The success of cryptocurrency in terms of allowing millions of successful and anonymous transactions with no hacking or corruption is evidence that the blockchain model works, at least for financial matters [77]. Migrating blockchain technology to healthcare informatics may be challenging, but it is likely inevitable [77]. The most immediately probable uses for blockchain in healthcare will be to create a decentralized network for electronic health records and data from clinical trials [77].

Decentralization prevents any one authority from having control of the system. Blockchain does not allow any data, once entered, to be altered. The blockchain paradigm makes all data anonymous, and any member participating in the system can see all things at any time. The predominant challenge with blockchain in healthcare is that it makes traceability impossible. Innovations, such as the Merkle tree, may be a good workaround for traceability [77]. A Merkle tree is a diagram in which every node or leaf of the tree bears a hash tag of the data block from which it came [78]; this permits subsequent data verification [79].

Blockchain can also be utilized together with AI, ML, and other machine algorithms by granting anonymous access to large amounts of data. Blockchain-based knowledge-sharing networks are already in existence and will likely increase [77]. MediLedger is a prescription drug supply chain service that uses blockchain, permitting users to check information about specific medications, including availability, expiration dates of supplies, and relevant legal issues [80]. Indeed, supply chain management may be an important area where blockchain applications offer unique benefits and does not pose major security challenges.

**Figure 1:**
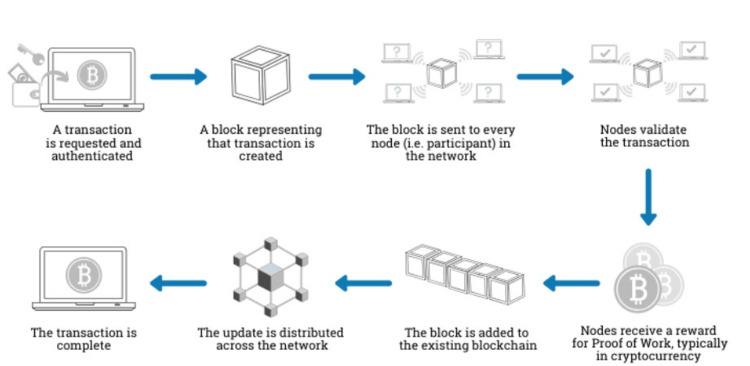
Entering a transaction or data into the blockchain system. Courtesy of the Department of Health and Human Services Cybersecurity Program

Electronic health records may soon benefit from blockchain technology, but they do offer unique considerations. While electronic medical records are familiar and accepted tools of the healthcare system, they are generated by each hospital, clinic, or practice. The result is a multi-part digital system fragmenting the patient’s experience over multiple organizations. There is no one single healthcare record that belongs to the patient; indeed, all electronic records are the property of their respective institutions [81]. Patient data that exist piecemeal over a series of disconnected digital networks create problems for the healthcare system, but there are currently no incentives for separate organizations to merge systems or even to foster compatibility across systems. Blockchain may be able to overcome that issue by consolidating all information, so that each provider has full access to all patient data [82]. The decentralized peer-to-peer blockchain network is sometimes called a “distributed ledger” system [83]. A decentralized system means that there is no one central authority but all peers have equal standing; it is the opposite of the top-down organizational pyramid, which consolidates power at the top [83]. Blockchain depends on “miners,” which are human users or nodes who compete with others to solve cryptographic problems to validate a particular block of information; if a miner can validate that information, the block gets added to the blockchain and there may be a reward for the blockchain miner [83] (see Figure 1).

Biometrics and medical identification

At a superficial level, new identity verification systems using biometrics have been introduced to healthcare as a way to help patients navigate around the sometimes cumbersome issue of user names and passwords to access medical portals [84]. In most systems, patients must present identification to obtain their own information: username/passwords for online access or presenting a driver’s license or passport when requesting information in person. New biometric identification systems would allow patients to gain instant access to their medical records from a smartphone without having to deal with passwords or other codes [84]. Biometrics are already in use for financial transactions; for instance, many banks allow for fingerprint or facial recognition on a smartphone.

For medical security, the risks associated with biometrics must be considered, because hacking is a real threat in all digital environments using multiple servers [85]. Problems with system integrity can compromise patient privacy and deny patients access to medical information they are entitled to access [86].

Biometric identification typically uses facial features, fingerprints, the iris, veins in the finger, or gait for identification, but other forms of physical identification are possible [87]. Novel systems requiring three different biometric identifications for added security are being developed and have achieved over 99% accuracy [88]. Security issues and data privacy have given rise of the transition from two-dimensional biometric systems to 3D biometrics [87]. New research is exploring the use of electroencephalogram (EEG) signals for biometrics [89].

Telehealth

The COVID-19 pandemic combined with the emergence of patients as healthcare consumers have both driven the uptake of telehealth options. The value of telehealth far exceeds emergency pandemic needs. Considering that approximately 20% of all Americans reside in rural areas (60 million citizens) where fewer than 10% of physicians practice, the emergence of telehealth also meets the need of providing care, especially specialized care, to people who do not have easy access to medical centers [90]. Telehealth services allow patients to consult with healthcare professionals using computer-based or smartphone communications methods.

The goal of telehealth, sometimes called virtual healthcare, is for its recognition and integration as a channel for healthcare delivery [91]. Telehealth may help reduce healthcare disparities besides offering convenience for many patients, including the disabled, rural citizens, those without domicile, or those for whom traveling back and forth to a clinic would represent a hardship [92]. The technologies of telehealth are not particularly revolutionary (e.g., videoconferencing), but the transition away from in-clinic to virtual care involves a large mental shift.

There are shortfalls to telehealth. For one thing, there is not a robust system in place for credentialing these services or to training providers to deliver care on such platforms [93]. There are also no uniform standards yet in place for robust quality assessment of these efforts [93]. Integrating telehealth into the standard practices and continuum of care within hospitals and clinics can be challenging [94]. While an abundance of telehealth options exist, healthcare providers may not always be able to refer their patients to appropriate options [95]. Reimbursement questions also remain open. Not all clinicians are comfortable using telehealth or with referring their patients to telehealth services.

By the same token, telehealth offers obvious benefits: it can provide more and more rapid healthcare delivery; it is convenient for patients and, if properly developed and leveraged, could be an important new revenue stream for many healthcare providers. Furthermore, it is crucial to see telehealth within the wider framework of digital healthcare-wearable devices that connect to telehealth applications. For example, a blood pressure spike occurring in a patient being monitored for hypertension could trigger a text alert to both patient and physician. The most formidable barrier to the full integration of telehealth into our healthcare system is no longer technology but imagination and will.

Real-time data analytics

AI and ML algorithms have been used to manage patient data in real-time data analytics, based in part on the model of real-time analysis pioneered by the National Aeronautics and Space Administration (NASA). Real-time data analytics using the NASA model would coordinate patient care across all departments in a large healthcare system, instantly sharing real-time information with other healthcare professionals on the continuum of care. Such a system of data analytics would allow care teams to form spontaneously, solve problems proactively, and manage the patient in real time.

Real-time data analytics is also capable of identifying and troubleshooting care delays and bottlenecks in the system. The system may be used to troubleshoot individual backlogs or delays, but it can also be deployed at the administrative level to facilitate appropriate staffing across the various departments at the facilities [96].

Another use of real-time data analytics are those systems that allow real-time risk assessment for surgical patients in terms of potential complications [97]. While many real-time analytics systems are advanced and complex, lightweight, user-friendly commercial products are being developed to facilitate specific real-time data usage by clinicians [98].

Privacy in the digital age

The robust protection of patient confidentiality and privacy remains a pillar of modern medicine [99]. Healthcare systems, clinics, hospitals, and all manners of clinicians understand the importance of guarding patient confidentiality [100]. However, many advanced technologies, platforms, AI, and deep learning were not designed for use in the healthcare system and, even when repurposed for healthcare use, remain in the hands of commercial enterprises. Even healthcare-benefitting technologies developed at academic institutions can wind up under private control. This opens a Pandora’s box of concerns, as private organizations typically do not offer and may not fully comprehend the level of privacy protections required by medical institutions [101]. Thus, patients may be informed in the hospital about Health Insurance Portability and Accountability Act (HIPAA) protections, only to have some of their private data be exported to third-party businesses. Technological progress is happening so rapidly it can easily outpace the demands of safeguarding patient data [101]. One example of trying to harness this proliferation of technology and impose privacy safeguards is offered by the FDA, who is now seeking to regulate AI companies rather than specific AI technologies [102]. The notion is that the technologies change faster than the speed of regulation.

A specific challenge in AI is that it remains a “black box” technology, meaning that the way the algorithm imports, analyzes, and interprets data is largely obscured from human observers. This means that erosions of security within the black box may be virtually invisible to humans as well [99]. AI uses algorithms to de-identify data, but new algorithms allow these systems to go back and re-identify data [101]. The fact that many AI technologies have been developed commercially and remain in commercial hands even when used for healthcare adds to the privacy conundrum.

Discussion

The Internet and digital technologies are in the process of changing the world, although it is not entirely clear yet how well the healthcare system will deal with these changes. Digital technologies offer exciting ways to tame huge amounts of data, error-proof many tedious and time-consuming processes, solve genetic puzzles, and take drudgery out of repetitive clinical tasks. Digital technologies will also ultimately empower patients to manage their own healthcare, do their own research, and monitor their own health. By the same token, digital technologies can lead to adverse effects, such as cyberchondria, or be misused. Digital advancements sometimes collide with privacy protections and the rate of technological innovation outpaces the speed of regulation.

Digital healthcare must be viewed holistically, although it is easy to get lost in its many moving parts. Telehealth, for example, is cost-effective and makes certain services more available to patients who might otherwise have limited to no access, but it should be seen as an adjunctive service and not a replacement for traditional in-clinic care. Important issues about data privacy or limitations to telehealth due to limited internet access, language barriers, or other concerns must be considered [103]. Wearables and smart apps can benefit individuals or preoccupy them; they can add to anxiety or empower health-conscious patients. Furthermore, digital devices encompass one aspect of health (e.g., number of steps and cardiac rhythms), which may not be a good surrogate for overall health [104].

Confidentiality and protecting health information must not be overlooked in the rush to comprehensive digital health programs, such as the extent to which data from smartphone apps or wearables are shared [105]. While such apps may disclose privacy risks in their terms and conditions, these documents can be confusing to laypeople and few patients read them anyway [105]. Few clinicians, let alone patients, understand the risks and frequency of third-party data-sharing by these apps. Even more esoteric is the so-called fourth-party data sharing, which can occur when data are monetized [105]. For example, a smartphone health app shares its data with a data broker; the broker is the third party and is not a healthcare organization. If that broker then sells the data to specific advertisers, the advertisers are the fourth party and are far removed from the original user. An example might be a bariatric patients, whose data are sold to a data broker (third party) who then brokers it to gyms and exercise programs (fourth party). These fourth parties offer ads to the patients because these commercial entities know who has had bariatric surgery. Such users may even be linked, so that sharing proceeds automatically. Data privacy is endangered across all fields, but the data privacy of people who, for example, have entered a marathon, are not as legally protected as those who have had certain medical procedures. In this world of targeted advertisements and data sharing, there seem to be no strong firewalls to protect health-related information. Indeed, a firm definition of what even constitutes health information is missing. For example, consumer-facing DNA testing organizations have been challenged over sharing such information with outsiders, including even law enforcement [106]. Genetic privacy laws are narrow and limited [106]. While these companies publish their privacy and data protection policies, these policies can be difficult even for attorneys to understand and can change without notice. Furthermore, data stored by these companies may be hacked or breached and companies may be acquired. These consumer-facing genetic testing companies have millions of users and may share anonymized data in projects with drug developers [107]. It should be noted that these companies are not health providers and are thus not regulated by federal health privacy rules, such as HIPAA [107]. This loophole may not be known to consumers or even healthcare providers. Digital trust in healthcare is yet to be well defined, but the two most obvious stakeholders in this issue are healthcare professionals and their patients with healthcare system administrators also in the running [108].

Digital health has tremendous potential but with it come great risks. Digital innovation is proceeding at a breakneck speed, faster than most busy clinicians can process these changes. This creates bottlenecks with regulatory organizations who, up until now, have devoted long periods of time into thorough examinations. Today, privacy regulations have to move faster than technology, but without ceding ground. One final key issue in digital health is that some of the more advanced technologies are enigmatic to healthcare professionals who, despite their considerable education and expertise, may not understand blockchain or deep learning algorithms. Clinicians need better education in these technologies, and the technology companies need better understanding of medical, regulatory, and privacy concerns. The technological gap between physicians and technology may be as wide as the privacy and ethics gap between technology innovators and healthcare.

This review has certain limitations. First, it is a narrative rather than systematic review. Second, it was meant to offer a round-up of new and important technologies and their potential clinical ramifications, but it did not offer a thorough evaluation of any of them. Third, technological advancements occur so rapidly, so this particular review must be considered in the time frame in which it was created.

## Conclusions

This is the beginning, not the culmination, of the digital revolution in healthcare. Many innovations seem destined to be long-lived, such as patient monitoring, AI, and virtual healthcare. Others may be more transient. The burgeoning amounts of patient-facing materials and resources accessible to patients can only empower health-literate patients and raising health literacy levels must be a priority as we enter this new era. The use of blockchain and other advanced technologies may be hard to imagine but could have powerful effects on healthcare, confidentiality, and privacy.
